# LncRNA HOTTIP regulates TLR4 promoter methylation by recruiting H3K4 methyltransferase MLL1 to affect apoptosis and inflammatory response of fibroblast‐like synoviocyte in rheumatoid arthritis

**DOI:** 10.1002/kjm2.12805

**Published:** 2024-02-16

**Authors:** Guan Wang, Yu‐Lin Xu, Xi‐Hai Zhang, Lian Tang, Yao Li

**Affiliations:** ^1^ Department of Orthopaedics The Affiliated Hospital of Southwest Medical University, Sichuan Provincial Laboratory of Orthopaedic Engineering Luzhou Sichuan China; ^2^ Laboratory Animal Center Southwest Medical University Luzhou Sichuan China

**Keywords:** HOXA transcript at the distal tip, mixed‐lineage leukemia 1, promoter methylation, rheumatoid arthritis, toll‐like receptor 4

## Abstract

Rheumatoid arthritis (RA) is a chronic autoimmune inflammatory disease, and the role of HOXA transcript at the distal tip (HOTTIP) in its pathogenesis remains underexplored. This study investigates the mechanism by which HOTTIP influences apoptosis and the inflammatory response of fibroblast‐like synoviocytes (FLS). An RA mouse model was established, and clinical scores were analyzed. Pathological changes in synovial tissues, bone mineral density (BMD) of the paws, serum tartrate‐resistant acid phosphatase (TRAP) activity, and TNF‐α and IL‐1β levels were assessed. FLS were transfected, and cell proliferation and apoptosis were examined. The RNA‐pull‐down assay determined HOTTIP's interaction with mixed‐lineage leukemia 1 (MLL1), while RNA immunoprecipitation assay measured HOTTIP expression pulled down by MLL1. The levels of MLL1 and toll‐like receptor 4 (TLR4) after MLL1 overexpression based on HOTTIP silencing were determined. Chromatin immunoprecipitation (ChIP) was performed with H3K4me3 as an antibody, followed by the evaluation of TLR4 expression. HOTTIP expression was elevated in RA mouse synovial tissues. Inhibition of HOTTIP led to reduced clinical scores, inflammatory infiltration, synovial hyperplasia, TRAP activity, and TNF‐α and IL‐1β levels, along with increased BMD. In vitro Interference with HOTTIP suppressed RA‐FLS apoptosis and inflammation. HOTTIP upregulated TLR4 expression by recruiting MLL1 to facilitate TLR4 promoter methylation. MLL1 overexpression reversed HOTTIP silencing‐mediated repression of RA‐FLS apoptosis. Activation of H3K4 methylation counteracted HOTTIP knockout, ameliorating the inflammatory response. HOTTIP regulates TLR4 expression by recruiting MLL1, leading to TLR4 promoter methylation, thereby suppressing RA‐FLS proliferation and inducing cell apoptosis and inflammatory response in RA.

## 1 INTRODUCTION

Rheumatoid arthritis (RA), characterized by systemic inflammation and persistent synovitis, remains a complex autoimmune disorder with unclear pathogenesis.^1^ Fibroblast‐like synoviocyte (FLS), crucial components derived from the synovial lining, play a pivotal role in joint destruction and abnormal inflammatory response in RA.^2^ However, the precise mechanisms underlying RA pathogenesis are still elusive,^3^ necessitating comprehensive exploration for therapeutic strategies.

Long non‐coding RNAs (lncRNAs), non‐protein‐coding transcripts exceeding 200 nt, emerge as key regulators in various disease processes, influencing gene expression by interacting with chromatin regulators and modulating RNAs.^4^ HOXA transcript at the distal tip (HOTTIP), an lncRNA, exerts regulatory control over diverse cellular processes, including apoptosis and cellular growth inhibition.^5^ Notably, HOTTIP has been identified as a crucial regulatory lncRNA in the etiology of knee osteoarthritis (OA).^6^ Importantly, studies indicate that lncRNA HOTTIP can recruit the methyltransferase mixed lineage leukemia 1 (MLL1), a specific methyltransferase that trimethylates histone H3K4.^7^ MLL1, a histone methyltransferase mediates enhanced trimethylation of histone H3 lysine 4 (H3K4me3) in the promoter region of toll‐like receptor 4 (TLR4), thereby facilitating TLR4 expression in myeloid cells.^8^ TLR4, an innate immune receptor for bacterial endotoxin, plays a critical role in inflammation, with documented expression in synovial tissues and peripheral blood monocytes of RA patients.^9^ Moreover, TLR4 activation in synovial fibroblasts contributes to joint destruction and chronic inflammation, highlighting its potential as a biomarker for RA treatment.^10^ This study aims to uncover the mechanism by which lncRNA HOTTIP regulates TLR4 promoter methylation by recruiting histone H3K4 methyltransferase MLL1, influencing apoptosis and inflammatory response in RA‐FLS, an aspect not yet explored in existing literature. Such exploration may unveil a novel molecular target with clinical implications for RA.

## 2 MATERIALS AND METHODS

### 2.1 Ethics statement

Ethical approval for all animal experiments were obtained from our University, and measures were taken to minimize the number of animals used and their discomfort.

### 2.2 Establishment of RA mouse models

Forty‐eight BALB/c mice (6–8 weeks old, weighing 22–27 g) were obtained from the Slake Jingda Animal Center (Changsha, Hunan, China) and housed in separate cages under a 12‐h light–dark cycle at 22–24°C and with free access to food and water. The mice were randomly divided into six groups (*n* = 8 each): Normal group, RA group, sh‐NC group, sh‐HOTTIP group, sh‐HOTTIP + DMSO group, and sh‐HOTTIP + PBIT group. RA modeling was performed as previously described,^2^ involving tail injections of 10 mg type II bovine collagen (Chondrex, Redmond, WA) with 100 μl of Freund's adjuvant and *Mycobacterium tuberculosis* (Biolead, Beijing, China). After 21 days, a booster injection was administered. Arthritis development was monitored based on visible swelling of feet and limbs, with specific criteria for ankle joint diameter and hind paw volume changes. Synovial hyperplasia was observed from weeks 1 to 4, followed by permanent articular cartilage degradation, manifested by chondrocyte mortality, fibrosis‐like structures, and cartilage degradation. Treatment commenced 3 weeks after initial immunization, with HOTTIP lentiviral interference and overexpression vectors, along with negative controls (NCs), injected via the tail vein.^11^ Lentivirus vectors (GenePharma, Shanghai, China) with a virus titer of 1.0 × 10^9^ TU/ml were used, with an injection volume of 3 μl.^12^ In the sh‐HOTTIP + PBIT group, 3 μl of 0.1% dimethyl sulfoxide (DMSO) or 5 mg/kg 2‐4(4‐methylphenyl)‐1,2‐benzisothiazol‐3(2H)‐one (PBIT) (HY‐101451, MCE, Monmouth Junction, NJ) was injected into mice intraperitoneally every 3 days for 16 consecutive days.

### 2.3 Clinical score

Mice were clinically scored for arthritis score and paw swelling on the 6th, 8th, 10th, 12th, 14th, and 16th days post‐treatment. The aggregate clinical score was calculated by summing the limb swelling score (0–3 per paw) and inflammation score (0–2 per mouse), with a maximum aggregate clinical score of 14. Inflammation scores were assigned as follows: 0, no inflamed digits; 0.5, 1–5 inflamed digits; 1, 6–10 inflamed digits; 1.5, 11–15 inflamed digits; and 2, 16 or more inflamed digits. Paw swelling scores were assigned as follows: 0, thickened ≤30%; 1, thickened >30%; 2, thickened >50%; and 3, thickened >80%.^2^

### 2.4 Micro‐computed tomography (Micro‐CT) analyses

Micro‐CT analyses were performed on anesthetized mice using 3% pentobarbital sodium (4 mg/kg). Bone mineral density (BMD) was examined using CTan software (Skyscan, Kontich, Belgium).^2^ Mice were anesthetized with an intraperitoneal injection of 3% pentobarbital sodium (50 mg/kg)^13^ and euthanized by cervical dislocation following blood collection from the posterior orbital venous plexus. Synovial tissues were separated, fixed in 4% neutral formaldehyde at 4°C overnight, decalcified with ethylenediamine tetraacetic acid (EDTA, Chaofeng Chemical, Suzhou, Jiangsu, China) for 2 weeks, embedded in paraffin wax, sectioned, and subjected to staining experiments.

### 2.5 Hematoxylin and eosin (HE) staining and inflammation scoring

Rear claws were fixed with 4% methanol for 24 h. After rinsing with graded ethanol (50%, 70%, 85%, 95%, and 100%, 2 h for each concentration), tissues were embedded in paraffin. Slices, 6 μm thick, were prepared at a 5° angle to the paraffin surface, followed by 5‐min each soak in 100%, 95%, 85%, and 75% graded ethanol, and 5‐min staining with hematoxylin and 2‐min eosin staining to assess joint pathology. Joint pathology was assessed by inflammation degree, conducted by three experts on a scale from 0 to 4: 0, no inflammation; 1 to 2, mild inflammation; 3, moderate inflammation; and 4, severe inflammation.^14^

### 2.6 Tartrate‐resistant acid phosphatase (TRAP) staining

The distribution of osteoclasts in synovial tissues was observed by TRAP staining. Paraffin sections of mouse synovial tissues were deparaffinized and washed with pure water. Sections were circled with an immunohistochemical pen, incubated with pure water at 37°C for 2 h, and then treated with TRAP working solution (PMK0467B, Bioprimacy, Wuhan, Hubei, China) at 37°C for 20–30 min in the dark. Nuclei were stained with hematoxylin, and sections were dehydrated, cleared, and sealed with neutral gum. TRAP‐positive osteoclasts, appearing wine red or light red, with light blue nuclei, were observed using a light microscope (OLYMPUS, Tokyo, Japan). Results were expressed as the percentage (%) of TRAP‐positive cells.

### 2.7 FLS culture and identification

RA‐FLS was generated by resuspending spliced RA synovial tissues in Dulbecco's modified Eagle's medium (DMEM) (Hyclone Laboratories, Losan, UT) supplemented with 10% fetal bovine serum (FBS) (Gibco Laboratories, Grand Island, NY) and transferring to tissue culture containers. Primary FLS migrated from biopsy tissues within the first 2 weeks. Monolayers were trypsinized and resuspended for propagation after reaching approximately 80% confluence. FLS from the third to the fifth generation were utilized for subsequent experiments.^15^

RA‐FLS were fixed, permeabilized, and incubated with Triton X‐100 for 30 min, followed by 8‐h incubation with primary antibody against vimentin (Bs‐8533R; Bioss, Beijing, China) at 4°C, and 1‐h incubation with secondary antibody goat anti‐rabbit (1:400, Abcam, Cambridge, MA). Hermetic tablets were visualized with a fluorescence microscope (BA410E; Motic, Xiamen, Fujian, China) following DAPI staining. Cell purity was assessed by flow cytometry (Becton, Dickinson and Company, Franklin Lakes, NJ). Cells at generations 3 to 5 were selected for following experiments, and FLS for negative control (NCFLS, HUMiCells010; iCell Bioscience, Shanghai, China) were incubated in DMEM supplemented with 10% FBS (sh30256.01; Hyclone).^16^

### 2.8 Cell transfection

HOTTIP small hairpin RNA (shRNA) sequences were designed, synthesized, and cloned into the shRNA vector U6/GFP/Neo plasmid (GenePharma). HOTTIP (oe‐HOTTIP) and MLL1 (oe‐MLL1) overexpression plasmid (pcDNA3.1‐ROCK2) was purchased from Generary Biotechnology (Shanghai, China). For transfection, 0.2 μg indicator plasmid and 0.4 μl liposome 3000 (Thermo Fisher, Waltham, MA) were mixed with 5 μl Opti‐MEM medium (Thermo Fisher) and introduced into FSL cells at approximately 70% confluence.^17^, ^18^, ^19^

### 2.9 Reverse transcription quantitative polymerase chain reaction (RT‐qPCR)

Total RNA was collected from cells and synovial tissues using the TRIzol reagent (Invitrogen, Calsbad, CA). RNA concentration and purity were assessed with a nanodrop 2000 micro‐ultraviolet spectrophotometer (1011U, nanodrop, Wilmington, DE). Reverse transcription was performed according to the protocols of the TaqMan MicroRNA Assays Reverse Transcription Primer (4427975, Applied Biosystems, Carlsbad, CA) for cDNA generation. Remaining RNA was reverse‐transcribed to cDNA using the PrimeScript RT Reagent Kit (RR047A, Takara, Otsu, Shiga, Japan). Primers for HOTTIP, MLL1, and TLR4 were designed and synthesized by TaKaRa company (Table 1). Real‐time fluorescence qPCR was conducted using the ABI7500 quantitative PCR instrument (7500, ABI, Foster City, CA), and the thermal cycling conditions included pre‐denaturation at 95°C for 10 min, followed by 40 cycles of denaturation at 95°C for 10 s, annealing at 60°C for 20 seconds, and extension at 72°C for 34 s. The relative expression levels of target genes (HOTTIP, MLL1, and TLR4) were calculated using the relative quantitative method (2^−ΔΔCT^ method) with glyceraldehyde‐3‐phosphate dehydrogenase (GAPDH) as an internal reference: ΔCt experimental group − ΔCt control group = ΔΔCt, Ct (target gene) − Ct (internal reference) = ΔCt.^20^ Each experiment was performed in triplicate.

**TABLE 1:** RT‐qPCR primer sequence.

Gene name	Primer sequence
HOTTIP	F: 5′‐AGGAGTACCCTGATGAGAT‐3′
	R: 5′‐GCCTTGGTGAGGTTTGAT‐3′
MLL1	F: 5′‐GCAGATTGTAAGACGGCGAG‐3′
	R: 5′‐GAGAGGGGGTGTTCCTTCCTT‐3′
TLR4	F: 5′‐AGTTGATCTACCAAGCCTTGAGT‐3′
	R: 5′‐GCTGGTTGTCCCAAAATCACTTT‐3′
GAPDH	F: 5′‐GGAGCGAGATCCCTCCAAAAT‐3′
	R: 5′‐GGCTGTTGTCATACTTCTCATGG‐3′

### 2.10 Western blot

Cells were lysed with 100 μl radioimmunoprecipitation assay (RIPA) lysis buffer (R0020, Solarbio Science & Technology, Beijing, China) containing 1 mmol/L of PMSF. The lysate was centrifuged at 12000*g* for 4 min following 30‐min ice incubation. The supernatant was sub‐packaged and frozen at −80°C. Protein concentration was determined using bicinchoninic acid kits (AR0146, Boster, Wuhan, Hubei, China), and the sample concentration was adjusted to 3 μg/μl. Protein samples were separated by 10% polyacrylamide gel electrophoresis and transferred to a polyvinylidene fluoride membrane (P2438, Sigma, St. Louis, MO) using the semi‐dry electro‐transfer membrane method. The membrane was blocked with 5% bovine serum albumin (10‐L16, Zhongsheng Likang Technology, Beijing, China) for 1 h at room temperature, and incubated with rabbit anti‐MLL1 (1:1000, ab234435, Abcam) and TLR4 (1:1000, ab13556, Abcam) overnight at 4°C. After three rinses with TBST (5 min each), the membrane was incubated with goat anti‐rabbit secondary antibody (1:2000, ab6721, Abcam) at room temperature for 1 h, followed by another three washes and development with chemiluminescence reagent, with glyceraldehyde‐3‐phosphate dehydrogenase (GAPDH) (1:5000, 60,004‐1‐Ig, Proteintech, Wuhan, Hubei, China) adopted as an internal reference. Imaging was performed using the Bio‐rad Gel Dol EZ imager (GEL DOC EZ IMAGER, Bio‐rad, Hercules, CA), and the target band was subjected to gray value analysis using Image J software (version 1.48, National Institutes of Health, Bethesda, MD). Each experiment was performed in triplicate.

### 2.11 Enzyme‐linked immunosorbent assay (ELISA)

ELISA kits from Zike Biological (Shenzhen, Guangdong, China) were employed to quantify interleukin‐1 (IL‐1β; ZK‐R3160) and tumor necrosis factor‐alpha (TNF‐α; ZK‐R3528) levels in the plasma homogenate and cells' supernatant. The ELISA kit (XYM1561A) used to measure serum tartrate‐resistant acid phosphatase (TRAP) levels were obtained from Xuanya Biotechnology (Shanghai, China). Quantification followed the provided instructions.^2^

### 2.12 Cell counting Kit‐8 (CCK‐8) assay

Cells seeded in 96‐well culture plates were cultured for 3 h after a 48‐h transfection period. Following incubation at 1 × 10^3^/well for 24, 48, and 72 h, 100 μl of fresh DMEM without FBS or penicillin–streptomycin solution (BC‐M‐014; Senbeiga Biotechnology, Nanjing, Jiangsu, China) was added to each well, followed by 1‐h incubation at 37°C. Optical density at 490 nm was determined using a spectrophotometer (BioTek Instruments, Winooski, VT). Each experiment was performed in triplicate, and the growth curve was plotted.^21^

### 2.13 Flow cytometry

Apoptosis was assessed by Annexin V‐fluorescein isothiocyanate (FITC)/propidium iodide (PI) double staining. After 48‐h transfection, cells were detached with 0.25% trypsin (EDTA‐free) (YB15050057, Yubo Biotechnology, Shanghai, China), collected tubes, and centrifuged, with the supernatant discarded. Cells were rinsed thrice with cold phosphate buffer saline (PBS), centrifuged, and the supernatant removed. Using the Annexin‐V‐FITC apoptosis detection kit (K201‐100, Biovision, San Francisco, CA), Annexin‐V‐FITC, PI, and 4‐(2‐hydroxyethyl)‐1‐piperazineethanesulfonic acid (HEPES) buffers were mixed into the Annexin‐V‐FITC/PI dye liquor (1:2:50). Cells were resuspended with 1 × 10^6^ cells per 100 μl of dye solution, and shaken evenly. After 15 min of incubation at room temperature, cells were mixed with 1 mL of HEPES buffer (PB180325, Procell, Wuhan, Hubei, China) and swayed uniformly. Fluorescence of FITC and PI was assessed by 525 and 620 nm band‐pass filters activated by 488 nm wavelengths, and apoptosis was evaluated. Each experiment was performed in triplicate.

### 2.14 RNA‐pull‐down assay

T7 RNA polymerase (Ambion, Austin, TX) transcribed the HOTTIP fragment in vitro. followed by DNase I (Qiagen, Hilden, Germany) and RNeasy Plus Mini Kit (Qiagen) treatment, and depuration with RNeasy Mini Kit (74104; Solabio Technology, Beijing, China). Purified RNA 3′ end was biotinylated using the biotin RNA labelling mixture (Ambion). Subsequently, 1 μg labeled RNA was warmed to 95°C in RNA structure buffer with 10 mmol/L MgCl_2_, 10 mmol/L Tris pH 7, and 0.1 mol/L KCl for 2 min, incubated for 3 min on ice, and later placed at room temperature for 30 min to form a suitable secondary structure. Moreover, cells (3 μg) were lysed for 1 h with the addition of cell lysis buffer (Sigma) at 4°C. The lysis buffer was centrifuged for 10 min at 12,000×*g* and 4°C, and the supernatant was collected and diverted to a RNase‐free centrifuge tube. Biotinylated RNA (400 ng) was mixed with RNA immunoprecipitation (RIP) buffer (500 μl) and incubated at room temperature with cell lysates for 1 h. Streptavidin magnetic beads were added to every binding reaction, and the samples were cultured for 1 h at room temperature. Eluted MLL1 protein was gauged by Western blot after rinsing with RIP buffer five times, adding 5 × loading buffer, and incubating at 95°C for 5 min.

### 2.15 RNA immunoprecipitation (RIP) assay

The interaction between HOTTIP and MLL1 protein was assessed using a RIP kit (millipore, Billerica, MA). Cells were gently washed with pre‐cooled PBS, and the supernatant was discarded. Cell lysis was performed with an equal volume of RIPA lysis buffer (P0013B, Beyotime Biotechnology, Shanghai, China) on ice for 5 min, followed by 10‐min centrifugation at 4°C and 12,000×*g* to obtain the supernatant. The cell extracts were co‐precipitated with antibody incubation. In each co‐precipitation reaction system, 50 μl of magnetic beads were washed and resuspended in 100 μl RIP Wash Buffer. Depending on the experimental group, 5 μg of antibody was added for incubation. The magnetic bead‐antibody complex was washed and resuspended in RIP Wash Buffer (900 μl), and incubated overnight with cell extract (100 μl) at 4°C. The samples were then placed on a magnetic base for magnetic bead‐protein complex collection. After detachment with proteinase K, RNA was extracted for subsequent PCR assay. Antibodies used for RIP were rabbit anti‐MLL1 (1:200, # 61702, Cell Signaling Technology, Boston, MA) mixed for 30 min at room temperature, with immunoglobulin G (1:100, ab109489, Abcam) as the negative control.^22^

### 2.16 Chromatin immunoprecipitation (ChIP) assay

Cells at 70–80% confluence were fixed and cross‐linked with 1% formaldehyde for 10 min at room temperature. Ultrasonic treatment was employed to fragment cells, cycling 15 times with each cycle consisting of 10 s of ultrasonication followed by 10‐s intervals. The fragmented cells were then centrifuged at 13,000 rpm and 4°C, and the supernatant was collected and divided into two tubes. These tubes were separately incubated with the negative control antibody rabbit anti‐immunoglobulin G (1:100, ab109489, Abcam) and the target protein‐specific antibody mouse anti‐H3K4me3 (1:100, ab8580, Abcam) overnight at 4°C. Protein Agarose/Sepharose (465464; Qiyue Biotechnology, Xi'an, Shaanxi, China) was used to precipitate endogenous DNA‐protein complexes, and the supernatant was aspirated and discarded after brief centrifugation. Following the washing of non‐specific complexes, decrosslinking was performed at 65°C overnight. Phenol/chloroform extraction and purification were done to recover DNA fragments, and qPCR was utilized to detect the content of TLR4 promoter fragments.^23^

### 2.17 Statistical methods

Data were processed using SPSS 21.0 (IBM Corp. Armonk, NY) and presented as mean ± SD for measurement data. Independent sample t‐tests were used for two‐group comparisons. One‐way analysis of variance (ANOVA) was applied for comparisons among groups, with Tukey's test. A value of *p* < 0.05 was considered statistically significant.

## 3 RESULTS

### 3.1 HOTTIP considered in RA mouse synovial tissues in vivo

**FIGURE 1:**
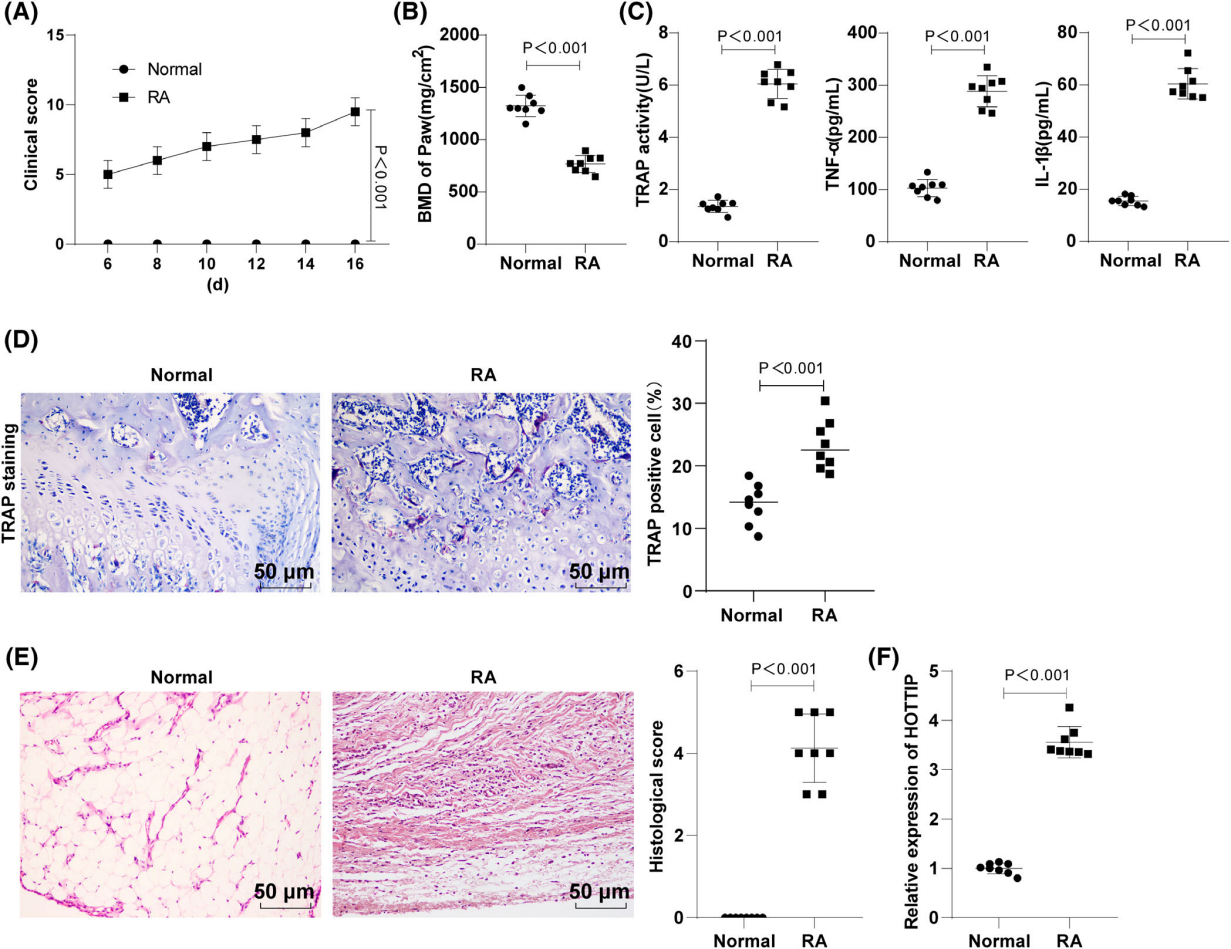
HOTTIP expression in synovial tissues of RA mice in vivo. (A) Clinical scores of mice after modeling were statistically analyzed; (B) MicroCT analysis of bone density in the paws of RA mice; (C) Determination of serum TRAP activity, and TNF‐α and IL‐1β levels using ELISA; (D) TRAP staining to observe the distribution of TRAP‐positive osteoclasts in synovial tissues; (E) HE staining to inspect the pathological changes in synovial tissues; (F) RT‐qPCR assay analyzing expression of HOTTIP in synovial tissues of RA mice. Data represented as mean ± SD, with independent sample *t*‐test for two‐group comparisons. *p* < 0.05 indicated a statistically significant difference. Panels A–F were from an in vivo experiment in mice. *N* = 8.

The investigation of HOTTIP's impact on RA mice began with the establishment of a mouse RA model, revealing significantly higher clinical scores in the RA group compared to the normal group (Figure 1A). MicroCT analysis of paw bone mineral density (BMD) indicated a consistent decrease in BMD in all RA mice compared to normal mice (Figure 1B). ELISA analysis of TRAP activity, IL‐1β and TNF‐α levels in serum demonstrated marked increases in the RA group (Figure 1C). TRAP staining revealed an elevated number of TRAP‐positive osteoclasts in the synovial tissues of RA mice (Figure 1D). HE staining further identified more severe synovial hyperplasia and inflammatory infiltration in the RA group relative to the normal group (Figure 1E). Moreover, as reflected by RT‐qPCR assay, the expression of HOTTIP was high in the synovial tissues of RA mice (Figure 1F).

### 3.2 Interference with HOTTIP mitigated tissue damage and inflammatory response in RA mice mice in vivo

**FIGURE 2:**
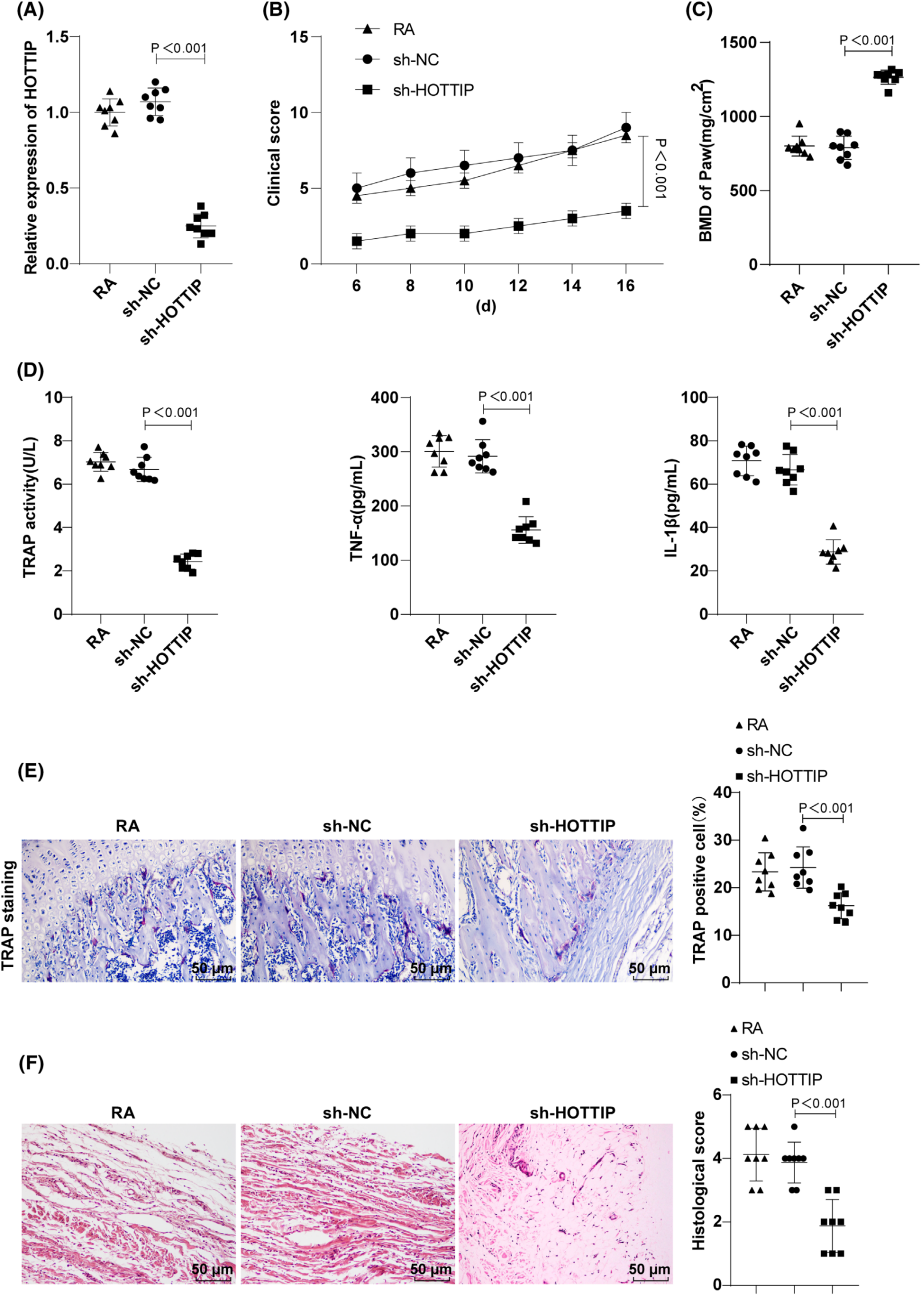
Impact of HOTTIP interference on inflammation and tissue damage in RA mice in vivo. (A) RT‐qPCR analysis of HOTTIP expression in synovial tissues of RA mice; (B) Clinical scores of mice after modeling were statistically analyzed; (C) MicroCT analysis of bone density in the paws of RA mice; (D) Determinations of serum TRAP activity, TNF‐α and IL‐1β levels using ELISA; (E) TRAP staining to observe the distribution of TRAP‐positive osteoclasts in synovial tissues; (F) HE staining to inspect pathological changes of synovial tissues. Data represented as mean ± SD, comparisons among groups conducted using one‐way ANOVA with Tukey's post‐test. *p* < 0.05 indicated the difference was statistically significant. Panels A–F were from an in vivo experiment in mice. *N* = 8.

To delve deeper into the influence of HOTTIP on RA, HOTTIP lentiviral interference vector was injected into mice, leading to a significant reduction in HOTTIP interference, as validated by RT‐qPCR (Figure 2A). Compared to the sh‐NC group, the sh‐HOTTIP group exhibited a notable decrease in clinical scores (Figure 2B). MicroCT analysis revealed a significant increase in BMD in the sh‐HOTTIP group (Figure 2C). ELISA results showed decreased TRAP activity, TNF‐α and IL‐1β levels in the sh‐HOTTIP group compared to the sh‐NC group (Figure 2D). TRAP staining exhibited a reduction in the number of TRAP‐positive osteoclasts after HOTTIP interference (Figure 2E). HE staining demonstrated alleviated symptoms of synovial hyperplasia and inflammatory infiltration in the sh‐HOTTIP group (Figure 2F). These results indicated that interfering with HOTTIP remarkably improved tissue damage and the inflammatory response in RA mice.

### 3.3 Interference with HOTTIP in vitro inhibited RA‐FLS inflammatory response and apoptosis

**FIGURE 3:**
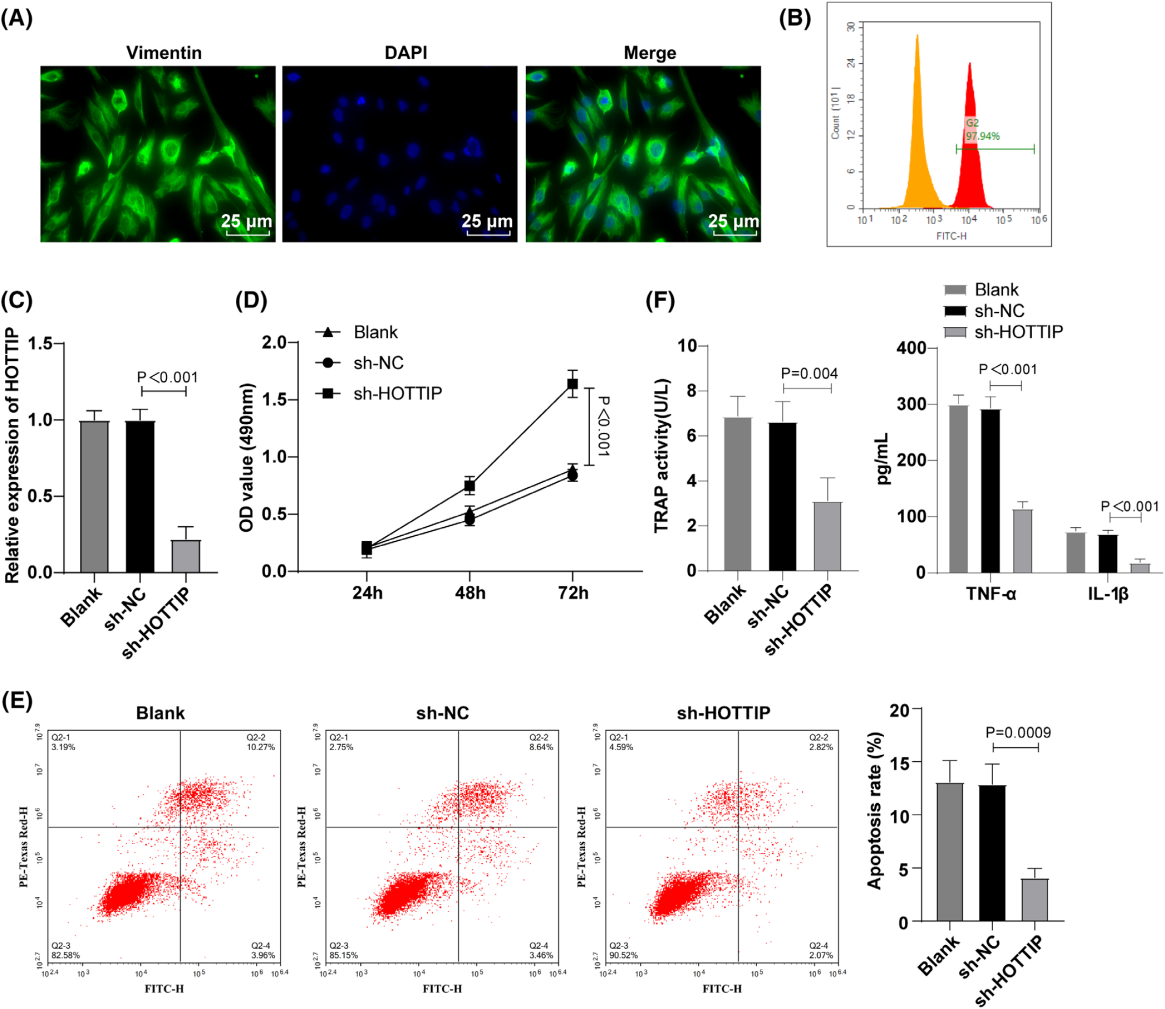
Effect of HOTTIP silencing on apoptosis and inflammation of RA‐FLS in vitro. (A) Immunofluorescence examination of vimentin expression in cells; (B) Flow cytometry detection of FLS cell purity; (C) RT‐qPCR measurement of HOTTIP interference efficiency in FSL cells; (D) CCK‐8 assay for cell proliferation; (E) Flow cytometry assessment of cell apoptosis; (F) ELISA for determining TRAP activity, TNF‐α and IL‐1β levels in cells. Data represented as mean ± SD, comparisons among groups conducted using one‐way ANOVA with Tukey's post‐test. *p* < 0.05 indicated a statistically significant difference. Panels A–F were performed in RA‐FLS cells. The experiment was repeated three times.

Immunofluorescence assay and flow cytometry confirmed the identity and purity of RA‐FLS cells, demonstrating the effectiveness of HOTTIP interference (Figure 3A, B). RT‐qPCR revealed reduced HOTTIP interference in FSL cells (Figure 3C). CCK‐8 assay and flow cytometry results indicated enhanced proliferative ability and reduced apoptotic rate in the sh‐HOTTIP group compared to the sh‐NC group (Figure 3D, E). ELISA analysis demonstrated decreased TRAP activity, TNF‐α, and IL‐1β levels in sh‐HOTTIP group compared to the sh‐NC group (Figure 3F). These findings suggested that interfering with HOTTIP in vitro restrained the apoptosis and inflammatory response of RA‐FLS.

### 3.4 HOTTIP up‐regulates TLR4 expression by recruiting MLL1 to enhance TLR4 promoter methylation in vitro

**FIGURE 4:**
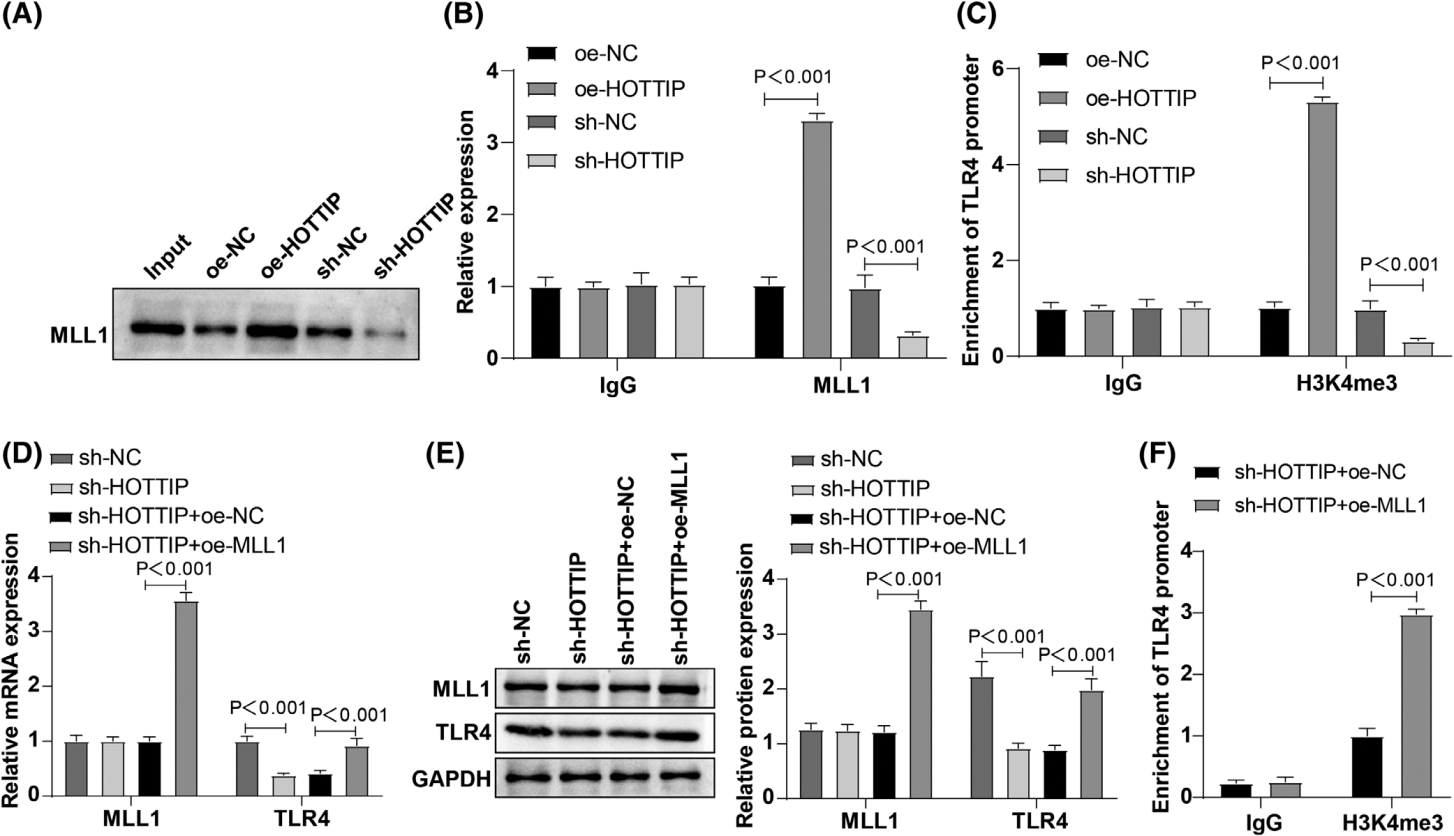
HOTTIP regulation of TLR4 expression through epigenetic mechanisms in vitro. (A) RNA‐pull‐down examination for HOTTIP recruitment of MLL1; (B) RIP measurement of HOTTIP pulled down by MLL1; (C) ChIP using H3K4me3 antibody, with qPCR verification of TLR4 expression after HOTTIP silencing or overexpression; (D, E) RT‐qPCR and Western blot for assessing expression levels of MLL1 and TLR4; (F) ChIP‐qPCR using H3K4me3 antibody for detection of TLR4 expression level. Data represented as mean ± SD, comparisons between two groups using independent sample *t*‐test, and among groups using one‐way ANOVA, followed by Tukey's test. *p* < 0.05 indicated a statistically significant difference. Panels A–F were performed in RA‐FLS cells. The experiment was repeated three times.

To elucidate the downstream mechanism of HOTTIP in RA‐FLS cells, we investigated whether HOTTIP could stimulate the trimethylation of H3K4 on TLR4 gene by recruiting methyltransferase MLL1, thereby promoting TLR4 transcription. RNA‐pull‐down and RIP assays (Figure 4A, B) revealed that HOTTIP could pull down MLL1, and its overexpression significantly increased MLL1 enrichment. Conversely, HOTTIP silencing markedly reduced its enrichment level on MLL1. ChIP assay results (Figure 4C) unveiled that HOTTIP overexpression notably increased H3K4me3‐enrichment on the TLR4 promoter, while HOTTIP knockout led to a prominent reduction in H3K4me3‐enrichment on the TLR4 promoter. RT‐qPCR and Western blot (Figure 4D, E) demonstrated that sh‐HOTTIP did not significantly affect MLL1 mRNA and protein levels. Moreover, the sh‐HOTTIP + oe‐MLL1 group showed notable elevations in TLR4 mRNA and protein levels compared to the sh‐HOTTIP + oe‐NC group. The ChIP experiment (Figure 4F) showed that the sh‐HOTTIP + oe‐MLL1 group exhibited distinctly higher H3K4me3 enrichment on the TLR4 promoter than the sh‐HOTTIP + oe‐NC group. Overall, HOTTIP recruitment of MLL1 encouraged H3K4 methylation, resulting in increased TLR4 expression.

### 3.5 Overexpression of MLL1 in vitro partially averted the inhibition effect of sh‐HOTTIP on apoptosis of RA‐FLS

**FIGURE 5:**
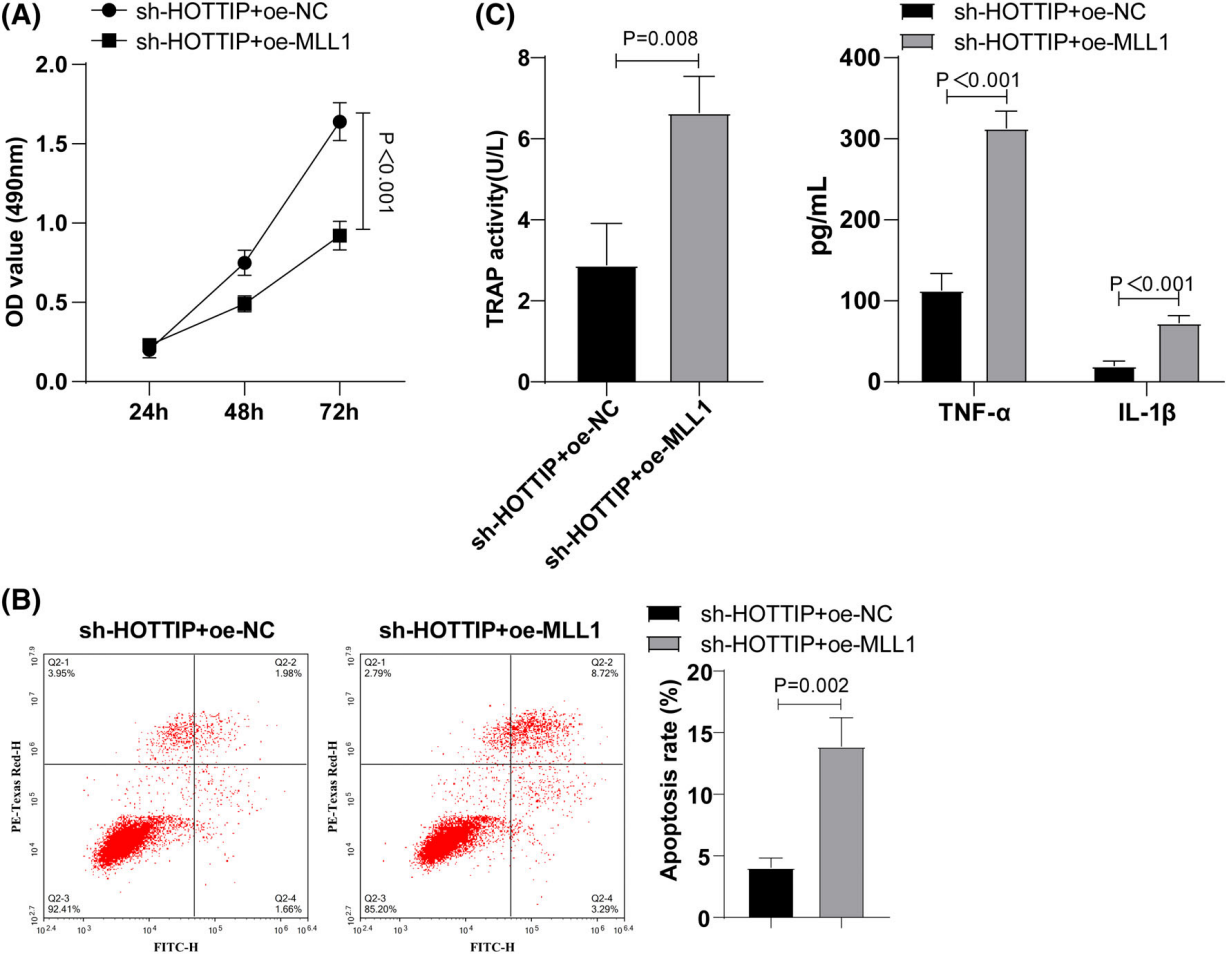
Overexpression of MLL1 partially deteriorates HOTTIP knockdown effects on inhibiting RA‐FLS apoptosis in vitro. (A) CCK‐8 detection of cell proliferation; (B) Flow cytometry assessment of cell apoptosis; (C) ELISA to determine inflammatory expression in cells. Data represented as mean ± SD, and independent sample *t*‐test used for two‐group comparisons. *p* < 0.05 indicated a statistically significant difference. Panels A–C were performed in RA‐FLS cells. The experiment was repeated three times.

To assess the role of lncRNA HOTTIP on RA‐FLS by regulating TLR4, FSL cells were divided into two groups (sh‐HOTTIP + oe‐NC and sh‐HOTTIP + oe‐MLL1). CCK‐8 and flow cytometry results (Figure 5A, B) indicated that, compared to the sh‐HOTTIP + oe‐NC group, the sh‐HOTTIP + oe‐MLL1 group displayed decreased proliferative ability and increased apoptotic rates. ELISA results (Figure 5C) showed that, relative to the sh‐HOTTIP + oe‐NC group, the sh‐HOTTIP + oe‐MLL1‐group exhibited increased TRAP activity, and TNF‐α and IL‐1β levels (Figure 5C). These findings suggested that overexpression of MLL1 partially annulled the suppressive effect of sh‐HOTTIP on apoptosis of RA‐FLS in vitro.

### 3.6 Activation of histone H3K4 methylation in vivo partially abrogated the improvement of inflammation in RA mice mediated by HOTTIP interference

**FIGURE 6:**
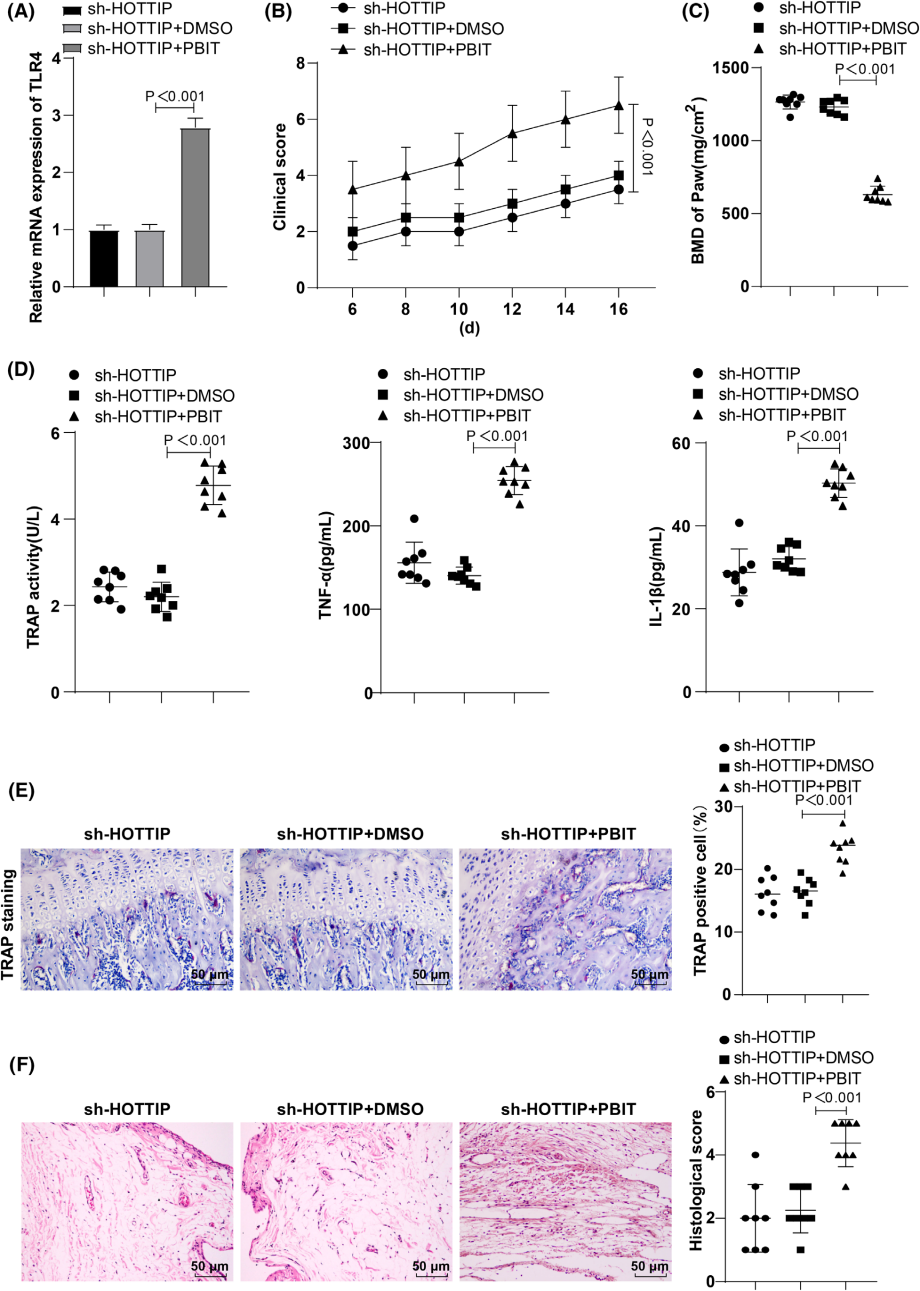
Activation of histone H3K4 methylation reverses the effect of HOTTIP interference on inflammatory response of RA mice in vivo. (A) RT‐qPCR analysis of TLR4 expression; (B) Statistical analysis of clinical scores in modeled mice; (C) MicroCT for analysis of mouse paw bone density; (D) ELISA determination of TRAP activity, TNF‐α and IL‐1β levels in serum; (E) TRAP staining observation of the distribution of TRAP‐positive osteoclasts in synovial tissues; (F) HE staining to inspect pathological changes of synovial tissues. Data represented as mean ± SD, and comparisons among groups performed using one‐way ANOVA with Tukey's test. *p* < 0.05 indicated a statistically significant difference. Panels A–F were from an in vivo experiment in mice. *N* = 8.

To investigate the impact of lncRNA HOTTIP on RA mice by regulating TLR4, the modeled mice were assigned to three groups (sh‐HOTTIP, sh‐HOTTIP + DMSO, and sh‐HOTTIP + PBIT, a H3K4me3 agonist). RT‐qPCR results (Figure 6A) showed an increase in TLR4 expression in the sh‐HOTTIP + PBIT group compared to the sh‐HOTTIP + DMSO group (Figure 6A). Clinical scores analysis revealed a significant increase in clinical scores in the sh‐HOTTIP + PBIT group versus the sh‐HOTTIP + DMSO group (Figure 6B). MicroCT analysis manifested a significant reduction in BMD in the sh‐HOTTIP + PBIT group compared to the sh‐HOTTIP + DMSO group (Figure 6C). ELISA results (Figure 6D) showed that IL‐1β and TNF‐α levels, and TRAP activity were markedly higher in the sh‐HOTTIP + PBIT group relative to the sh‐HOTTIP + DMSO group (Figure 6D). TRAP staining revealed an augmentation in the quantity of TRAP‐positive osteoclasts in the synovial tissues (Figure 6E). HE staining illustrated that the addition of PBIT sharpened the symptoms of inflammatory infiltration and synovial hyperplasia (Figure 6F). These results implied that the activation of histone H3K4 methylation partially reversed the improvement of inflammatory response made by the obstruction of sh‐HOTTIP in RA mice.

## 4 DISCUSSION

Rheumatoid Arthritis (RA) poses a significant threat, leading to bone damage, cartilage degradation, and disability.^24^ With an incidence ranging from 0.5% to 1.0% across populations,^25^ the intricate mechanisms driving its pathogenesis involve the dysregulation of long non‐coding RNAs (lncRNAs), impacting DNA methylation and histone regulation.^26^, ^27^ Mounting evidence supports the idea that modulating HOTTIP could be a promising strategy in treating RA.^28^ Our emphasizes the role of HOTTIP in TLR4 promoter methylation, resulting in elevated TLR4 expression through the recruitment of MLL1. This, in turn, inhibits the proliferation of RA Fibroblast‐Like Synoviocytes (RA‐FLS) and exacerbates inflammatory response and cell apoptosis.

RA patients face an increased risk of fractures accompanied by a well‐documented decline in bone mineral density (BMD).^29^ Given the chronic inflammatory nature of RA, pro‐inflammatory cytokines, including TNF, IL‐1, IL‐6, and IL‐18, play pivotal roles in its pathogenesis.^30^, ^31^ Notably, the IL‐1β signal is particularly significant in the pathogenesis of inflammatory arthritis, especially RA.^32^ Emerging evidence underscores the substantial involvement of lncRNAs in RA pathogenesis.^33^ For example, lncRNA GAS5 has been shown to limit RA by upregulating PDK4 and downregulating miR‐361‐5p.^34^ In the context of RA, HOTTIP has been identified as a crucial regulator lncRNA in knee Osteoarthritis (OA) pathogenesis.^35^ Previous research indicates that HOTTIP, through lncRNA Chip analysis, leads to synovial hyperplasia in RA and intensifies its severity, contributing to destructive effects in RA through accelerated inflammation.^28^ Our study aligns with these observations, revealing increased levels of IL‐1β and TNF‐α, heightened TRAP activity, clinical scores, and decreased BMD in the paws, along with exacerbated inflammatory infiltration, synovial hyperplasia, and elevated HOTTIP expression in RA mice. Collectively, our findings provide initial insights into the upregulation of HOTTIP in the synovial tissues of RA mice.

Fibroblast‐like synoviocytes (FLS) represents a unique cell population in synovial tissues, and their apoptosis in FLS is intricately linked to RA development.^36^ By knocking down HOTTIP in vivo and in vitro, we observed reduced levels of TNF‐α and IL‐1β, clinical scores, TRAP activity, synovial hyperplasia, and inflammatory infiltration, coupled with increased BMD in RA mice. This aligns with in vitro results indicating enhanced proliferative ability and suppressed TRAP activity, apoptotic rate, IL‐1β, and TNF‐α levels in FLS. This echoes findings in an asthmatic mouse model where downregulating HOTTIP reduced inflammation, resulting in decreased secretion of IL‐4, IgE, IL‐5, and IL‐13, and reduced infiltration of inflammatory cells.^37^ Moreover, decreased HOTTIP significantly suppressed the release of pro‐inflammatory factors IL‐1β and IL‐8 in macrophages during acute gouty arthritis.^38^ These novel insights suggest, for the first time, that interference with HOTTIP attenuates tissue damage and inflammatory responses in RA mice in vivo, thereby suppressing RA‐FLS apoptosis and inflammation in vitro.

The interplay between HOTTIP and MLL1 is pivotal, as it facilitates H3K4me3, a histone modification closely associated with gene expression regulation.^7^ In the context of TLR4, a receptor implicated in RA pathology,^39^ we delved deeper into the downstream mechanisms of HOTTIP in RA. Our results demonstrated that HOTTIP interacts with MLL1, and its overexpression enhances the enrichment of MLL1 and H3K4me3 on the TLR4 promoter. Conversely, the depletion of HOTTIP results in decreased enrichment. Notably, augmenting MLL1 expression on top of HOTTIP knockout led to elevated TLR4 expression and increased H3K4me3 on the TLR4 promoter. These findings substantiate our hypothesis that HOTTIP recruits MLL1, fostering H3K4 methylation, and subsequently promoting TLR4 expression. To delve deeper into the impact of lncRNA HOTTIP on RA‐FLS through TLR4 regulation, we overexpressed MLL1 following HOTTIP knockout in FLS. This intervention resulted in diminished proliferative ability, increased apoptotic rate, heightened TRAP activity, and elevated TNF‐α and IL‐1β levels. Parallel in vivo experiments further validated these outcomes, showcasing that the activation of H3K4me3 partially counteracted the alleviation of inflammatory response brought about by HOTTIP silencing.

In summary, our study provides robust support for the notion that HOTTIP stimulates TLR4 promoter methylation, consequently upregulating its expression by recruiting MLL1. This process restrains the proliferation of RA‐FLS while promoting cell apoptosis and inflammatory responses. However, it's imperative to note that this study only simply revealed the possible molecular mechanism of HOTTIP contributes to the onset and progression of RA in vivo and in vitro by regulating TLR4 promoter methylation. The specific sites for HOTTIP recruitment of MLL1 and MLL1‐mediated TLR4 promoter methylation are yet to be explored comprehensively. Hence, our future research direction involves a meticulous investigation to pinpoint the precise sites for MLL1 recruitment by HOTTIP and the subsequent MLL1‐mediated TLR4 promoter methylation.

## CONFLICT OF INTEREST STATEMENT

All authors declare no conflict of interest.
